# An international multi-center investigation on the accuracy of radionuclide calibrators in nuclear medicine theragnostics

**DOI:** 10.1186/s40658-020-00338-3

**Published:** 2020-11-23

**Authors:** Clarita Saldarriaga Vargas, Matthias Bauwens, Ivo N. A. Pooters, Stefaan Pommé, Steffie M. B. Peters, Marcel Segbers, Walter Jentzen, Andreas Vogg, Floris H. P. van Velden, Sebastiaan L. Meyer Viol, Martin Gotthardt, Felix M. Mottaghy, Joachim E. Wildberger, Peter Covens, Roel Wierts

**Affiliations:** 1 Radiation Protection Dosimetry and Calibrations, Belgian Nuclear Research Centre (SCK CEN), Mol, Belgium; 2 In vivo Cellular and Molecular Imaging, Vrije Universiteit Brussel, Jette, Belgium; 3 Department of Radiology and Nuclear Medicine, Maastricht University Medical Center, P.O. Box 5800, 6202 AZ Maastricht, The Netherlands; 4 European Commission, Joint Research Centre (JRC), Geel, Belgium; 5 Department of Radiology, Nuclear Medicine and Anatomy, Radboudumc, Nijmegen, The Netherlands; 6 Department of Radiology and Nuclear Medicine, Erasmus MC, Rotterdam, The Netherlands; 7 Department of Nuclear Medicine, University of Duisburg-Essen, Essen, Germany; 8 Department of Nuclear Medicine, University Hospital RWTH Aachen University, Aachen, Germany; 9 Department of Radiology, Leiden University Medical Center, Leiden, The Netherlands; 10 Department of Radiology and Nuclear Medicine, University Medical Center Utrecht, Utrecht, The Netherlands

**Keywords:** Activity measurement, Radionuclide calibrator, Accuracy, Theragnostics

## Abstract

**Background:**

Personalized molecular radiotherapy based on theragnostics requires accurate quantification of the amount of radiopharmaceutical activity administered to patients both in diagnostic and therapeutic applications. This international multi-center study aims to investigate the clinical measurement accuracy of radionuclide calibrators for 7 radionuclides used in theragnostics: ^99m^Tc, ^111^In, ^123^I, ^124^I, ^131^I, ^177^Lu, and ^90^Y.

**Methods:**

In total, 32 radionuclide calibrators from 8 hospitals located in the Netherlands, Belgium, and Germany were tested. For each radionuclide, a set of four samples comprising two clinical containers (10-mL glass vial and 3-mL syringe) with two filling volumes were measured. The reference value of each sample was determined by two certified radioactivity calibration centers (SCK CEN and JRC) using two secondary standard ionization chambers. The deviation in measured activity with respect to the reference value was determined for each radionuclide and each measurement geometry. In addition, the combined systematic deviation of activity measurements in a theragnostic setting was evaluated for 5 clinically relevant theragnostic pairs: ^131^I/^123^I, ^131^I/^124^I, ^177^Lu/^111^In, ^90^Y/^99m^Tc, and ^90^Y/^111^In.

**Results:**

For ^99m^Tc, ^131^I, and ^177^Lu, a small minority of measurements were not within ± 5% range from the reference activity (percentage of measurements not within range: ^99m^Tc, 6%; ^131^I, 14%; ^177^Lu, 24%) and almost none were outside ± 10% range. However, for ^111^In, ^123^I, ^124^I, and ^90^Y, more than half of all measurements were not accurate within ± 5% range (^111^In, 51%; ^123^I, 83%; ^124^I, 63%; ^90^Y, 61%) and not all were within ± 10% margin (^111^In, 22%; ^123^I, 35%; ^124^I, 15%; ^90^Y, 25%). A large variability in measurement accuracy was observed between radionuclide calibrator systems, type of sample container (vial vs syringe), and source-geometry calibration/correction settings used. Consequently, we observed large combined deviations (percentage deviation > ± 10%) for the investigated theragnostic pairs, in particular for ^90^Y/^111^In, ^131^I/^123^I, and ^90^Y/^99m^Tc.

**Conclusions:**

Our study shows that substantial over- or underestimation of therapeutic patient doses is likely to occur in a theragnostic setting due to errors in the assessment of radioactivity with radionuclide calibrators. These findings underline the importance of thorough validation of radionuclide calibrator systems for each clinically relevant radionuclide and sample geometry.

**Supplementary Information:**

The online version contains supplementary material available at 10.1186/s40658-020-00338-3.

## Introduction

In the last decades, the application of personalized molecular radiotherapy using theragnostics has gained a lot of interest in nuclear medicine [1, 2]. Theragnostic approaches aim to optimize molecular radiotherapy for individual patients using pre-therapeutic diagnostic imaging. In particular, assessment of the therapeutic absorbed dose to malignant tissue and to organs at risk based on these images facilitates a personalized therapeutic activity approach. These approaches require accurate quantification of the activity administered to patients both in diagnostic and therapeutic applications. Accurate activity calibration of radionuclide imaging equipment such as SPECT and PET cameras is also essential in theragnostics, to enable an accurate estimation of radiopharmaceutical uptake in patient tissues.

In practice, radionuclide activity calibrators are used to measure the radiopharmaceutical activity to be administered to patients and are often the reference instrument for calibrating SPECT and PET systems. Radionuclide calibrators are typically provided with factory-set calibration factors for a variety of clinically relevant radionuclides. Usually, the calibration factors are calculated from energy-dependent sensitivity curves, determined experimentally on a dedicated reference device using well-calibrated traceable sources in standard containers [3]. In-factory calibration of medical devices is usually limited to a small subset of (long-lived) radionuclides to ensure proper response of each device with respect to the reference device. However, due to manufacturing tolerances in device specifications, variations in response among radionuclide calibrators of same model can occur, particularly in the low photon-energy range, which is generally not tested in the factory. Moreover, sample geometries used in clinical practice differ in shape, size, material, and filling volume from the standard container geometries used for activity calibrations. Since radionuclide calibrator measurements are sensitive to changes in system and sample measurement geometry [4, 5], the validity of generic factory-set calibration factors is not guaranteed for clinically used radionuclides and sample geometries.

Therefore, several international guidelines recommend a thorough validation of radionuclide calibrator accuracy for all clinically used radionuclides and sample geometries during acceptance testing [6–8]. These guidelines typically recommend a measurement accuracy of ± 5–10% for diagnostic and ± 5% for therapeutic radionuclides. However, although practice varies widely across Europe, more often than not radionuclide calibrators are clinically implemented without such validation due to a lack of available certified activity standards of (short-lived) clinically used radionuclides, expertise, and time/costs required to perform this validation. In fact, a multi-center study investigating the radionuclide calibrator measurement accuracy among 15 Belgian hospitals performed between 2013 and 2015 revealed that none of the participating centers assessed the accuracy of clinically used radionuclides [9].

Several studies [9–15] have reported on the measurement accuracy of various individual diagnostic and therapeutic radionuclides, and demonstrated large measurement deviations (> ± 10%), particularly for ^111^In, ^68^Ga, ^123^I, and ^90^Y. However, no study has reported on the combined error of radiopharmaceutical activity measurements with radionuclide calibrators in the increasing application of personalized molecular radiotherapy based on a theragnostic approach. Therefore, we performed an international multi-center study on the clinical measurement accuracy of 32 radionuclide calibrators (7 different types from 4 vendors) for a comprehensive set of theragnostic radionuclides: imaging tracers ^99m^Tc, ^111^In, ^123^I, and ^124^I, and their therapeutic companions ^90^Y, ^177^Lu, and ^131^I. Additionally, the combined deviation of activity measurements in a theragnostic setting was evaluated for 5 clinically relevant theragnostic pairs: ^131^I/^123^I and ^131^I/^124^I, which are used mostly for treatment of thyroid disorders such as differentiated thyroid cancer and hyperthyroidism; ^177^Lu/^111^In and ^90^Y/^111^In, used for peptide receptor radionuclide therapy of neuroendocrine neoplasms and prostate cancer; and ^90^Y/^99m^Tc, used in the treatment of liver tumors and metastases with ^90^Y microspheres [1, 2, 16].

## Methods

### Stock solution preparation

The radionuclides were obtained from various suppliers: [^99m^Tc]-NaTcO_4_, [^123^I]-NaI, and [^131^I]-NaI from GE Healthcare (Eindhoven, The Netherlands); [^124^I]-NaI from BV Cyclotron VU (Amsterdam, The Netherlands); [^177^Lu]-LuCl_3_ from IDB Holland (Baarle-Nassau, The Netherlands); and [^111^In]-InCl_3_ and [^90^Y]-YCl_3_ from Curium (Petten, The Netherlands).

[^177^Lu]-LuCl_3_, [^111^In]-InCl_3_, [^90^Y]-YCl_3_, [^131^I]-NaI, and [^124^I]-NaI stock solutions and samples were prepared within 24 h of the first day of the intercomparison measurements, which took place over three consecutive days. Due to their shorter half-life, [^99m^Tc]-NaTcO_4_ and [^123^I]-NaI solutions were prepared at each measurement day. For each radionuclide, a stock solution was prepared with approximately 10 MBq mL^−1^ on the first measurement day. Stock solutions were prepared using sterile water (Baxter, The Netherlands) in a borosilicate glass container and immediately after preparation dispensed into samples to avoid precipitations.

### Evaluation of radionuclidic impurities

Each stock solution was checked for radionuclidic impurities by high-resolution gamma-ray spectrometry using a high-purity germanium detector (GR1018; Mirion Technologies, Georgia, USA) as described in the supplemental material. No short- or long-lived radionuclidic impurities were found for ^99m^Tc, ^111^In, ^131^I, and ^90^Y. For ^123^I and ^124^I, trace amounts of ^125^I were observed with a maximum radionuclidic impurity of 0.030% and 0.037%, respectively. For ^177^Lu, trace amounts of ^177m^Lu were observed with a maximum radionuclidic impurity of 0.017%. Minimum detectable activities of potential impurities not detected (^99^Mo, ^114m^In, ^121^Te, ^88^Y) and the effect of (potential) impurities on a radionuclide calibrator are reported in the supplemental material (Table S1) [17].

### Determination of reference activity

The reference (true) activity concentration of each stock solution was determined by the Belgian Nuclear Research Centre (SCK CEN) (Mol, Belgium) in collaboration with the Joint Research Centre (Geel, Belgium), which is specialized in primary and secondary standardization of radioactivity [18]. Reference activity measurements were performed using two secondary standard ionization chambers: a Fidelis (Southern-Scientific, Henfield, UK) and an ISOCAL-III (Vinten Instruments, UK). The latter is consistent with radioactivity standards from the JRC [9]. Both chambers are of the same design and use calibration factors traceable to the primary standards of activity of the UK National Physical Laboratory (NPL).

From each stock solution, three 10-mL type 1+ Schott vials (SCHOTT AG Pharmaceutical Systems, Mainz, Germany) [19] were filled with 4 mL of solution (calibration geometry specified for the Fidelis), and their activities were assayed in both reference chambers. With the exception of ^90^Y, the reference activity of each Schott vial was determined from the mean of the activities measured with both the Fidelis and the ISOCAL, and the gravimetrically determined mass of stock solution in the vial. All activity measurements were corrected for background signal and for radioactive decay to a common reference time using the half-life values published in the NuDat database version 2.8 [20]. Additionally, before determination of the average value, the activity measurements were corrected for linearity, radionuclide impurities (significant only for (^177m^Lu/)^177^Lu measurements), and deviations in response against the NPL master chamber (see supplementary Table S2). For the latter correction, radionuclide- and chamber-dependent correction factors were estimated from the NPL acceptance testing data of each system (corrections < 1.1% for the gamma emitters and 14.5% for the ^90^Y Fidelis measurements), as described in the supplementary data [21].

With the exception of ^90^Y, the Fidelis and ISOCAL systems agreed within ± 0.7% in Schott vial activity measurements. For ^90^Y, however, a difference in response of approximately 10% was observed between both systems. On the basis of this discrepancy and the lack of experimental data to correct the response of the ISOCAL against the NPL master chamber for pure beta emitters, the reference activity concentration of the ^90^Y stock solution was derived from activity measurements with the Fidelis only. The reference activity concentration of the radionuclide stock solution was then determined as the mean of the activity concentrations from the three Schott vials. The expanded uncertainty (95% confidence level) in the reference activity concentrations of the stock solutions was 2.0% for ^99m^Tc, 1.7% for ^111^In, 2.2% for ^123^I, 2.0% for ^124^I, 1.1% for ^131^I, 1.2% for ^177^Lu, and 6.9% for ^90^Y (see supplementary Table S3).

### Sample preparation

From each stock solution, a set of four samples comprising two different clinical containers each with two filling volumes were prepared: two 3-mL Luer-lock syringes (Terumo Europe, Leuven, Belgium) filled with 1 mL and 3 mL of solution, and two 11-mL TechneVial glass vials (Curium, Petten, The Netherlands) filled with 1 mL and 10 mL of solution. Each syringe was sealed with a combi-stopper (Braun, The Netherlands). The content mass of each sample was verified gravimetrically, by weighing the sample before and after filling with an analytical balance (XS105DU/M; Mettler-Toledo, Tiel, The Netherlands). The reference activity (*A*_ref_) of each sample was calculated by multiplying the content mass with the stock solution reference activity concentration. As the uncertainty in sample mass measurements was negligible compared to the uncertainty in radioactivity concentration, the relative uncertainty of the sample reference activity (*u*_ref_) was approximately equal to the relative uncertainty of the stock solution activity concentration.

Due to transport logistics, for one hospital, separate sets of samples (a 3-mL syringe filled with 3 mL and a TechneVial filled with 10 mL of stock solution) were prepared for all radionuclides.

### Clinical activity measurements

Sample measurements were performed on a total of 32 radionuclide calibrator systems of 8 university hospitals located in the Netherlands, Belgium, and Germany. Of all systems, 4 were manufactured by Capintec Inc (Florham Park, USA), 11 by former MED Nuklear-Medizintechnik (now Nuvia Instruments, Dresden, Germany), 1 by PTW-Freiburg (Freiburg, Germany), and 16 by former Veenstra Instruments (now Comecer Netherlands, Joure, The Netherlands) (see supplemental Table S4).

If applicable, measurements were performed using hospital-specific calibration settings and sample geometry corrections. Otherwise, standard factory settings were used (see supplementary Tables S5-S11). The standard (automatic) measurement (averaging) time of the calibrator was used. Three activity readings (*n* = 3) were taken sequentially, without moving the sample, at intervals of several seconds (dependent on observed system response time). The calibrator reading was left to settle (typically for about 15 to 30 s) before the first reading was taken. The range of the sample activities at the moment of clinical measurements is indicated in Table 1. Each measurement was corrected for background signal and radioactive decay. For each measurement triplet, the average net activity (*Ā*_*m*_) and standard deviation (*SD*) were calculated. The statistical measurement uncertainty (*u*_*m*_) was estimated at the 95% confidence level (coverage factor *k* = 4.30 for a *t*-distribution with two (*n* − 1) degrees of freedom), as follows:

1 $$ {u}_m=\frac{k\bullet SD}{{\overline{A}}_m\bullet \sqrt{n}} $$

**Table 1:** Sample reference activities (minimum–maximum (25th percentile)) at the moment of clinical activity measurements

Radionuclide	Minimum ***A***_**ref**_–maximum ***A***_**ref**_ (25th percentile) (MBq)			
	Syringe 1 mL	Syringe 3 mL	Vial 1 mL	Vial 10 mL
^99m^Tc	3.9–18.5 (6.7)	10.3–44.3 (18.1)	4.3–18.0 (7.1)	34.1–147.8 (58.6)
^111^In	5.3–9.5 (5.7)	14.4–25.6 (15.4)	5.9–10.5 (6.3)	48.1–85.6 (51.4)
^123^I	6.7–17.6 (9.6)	17.6–47.5 (25.2)	7.8–19.9 (11.1)	60.7–161.6 (86.6)
^124^I	6.8–10.3 (7.1)	10.8–25.4 (17.4)	7.9–12.0 (8.3)	35.0–85.7 (58.6)
^90^Y	17.3–38.8 (18.7)	20.4–94.3 (45.2)	14.4–32.4 (15.6)	67.4–312.4 (149.7)
^131^I	10.8–13.4 (11.1)	20.4–31.5 (26.0)	13.1–16.2 (13.4)	66.8–105.7 (87.3)
^177^Lu	8.9–12.5 (9.2)	17.8–34.2 (25.2)	10.4–14.4 (10.7)	57.9–111.4 (82.0)

Net activities were not corrected for the presence of radionuclidic impurities (if any).

### Evaluation of performance

#### Individual radionuclides

The radionuclide calibrator measurement accuracy was determined as the percentage deviation of the average measured activity *Ā*_*m*_ with respect to the sample reference activity *A*_ref_.

For each radionuclide and sample geometry, the typical accuracy and reliability of activity measurements were described in terms of the median and the inter-quartile range (IQR) values of the measurement percentage deviations of all systems pooled together. Similarly, these metrics were used to assess the manufacturer dependence of measurement accuracy and inter-system variability. Sample geometry effects were evaluated by comparing the measurement deviations of the syringe and vial samples with similar filling volume (syringe 1 mL vs vial 1 mL, syringe 3 mL vs vial 10 mL).

#### Theragnostic pairs

Finally, since patient tissue doses are proportional to the amount of therapeutic activity administered and in a theragnostic approach the amount of therapeutic activity is based on diagnostic imaging, the combined systematic percentage deviation (bias) that would be associated to therapeutic doses (*E*_*D*_) was calculated for the theragnostic pairs ^131^I/^123^I, ^131^I/^124^I, ^177^Lu/^111^In, ^90^Y/^99m^Tc, and ^90^Y/^111^In, as follows:

2 $$ {E}_D=\left[\frac{{\left({\overline{A}}_m/{A}_{\mathrm{ref}}\right)}_{\mathrm{therapy}}}{{\left({\overline{A}}_m/{A}_{\mathrm{ref}}\right)}_{\mathrm{imaging}}}-1\right]\bullet 100\% $$

## Results

### Data analysis

**Fig. 1:**
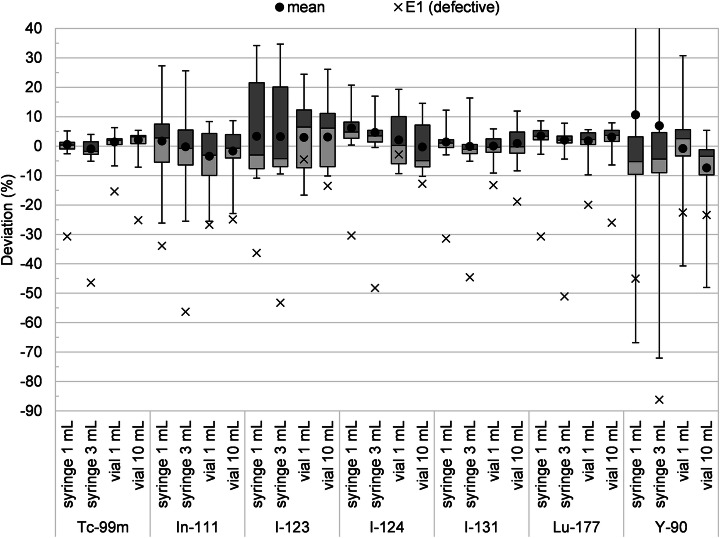
Box-whisker plots and mean values of the percentage deviations of all activity measurements used for analysis, for each radionuclide sample configuration tested (^90^Y whisker limits not shown: syringe 1 mL 423.9%, syringe 3 mL -383.6%). Additionally, the percentage deviations from defective measurements excluded from the analysis and box-whisker plots are shown as data points

In total, 32 radionuclide calibrator systems were investigated. If no calibration setting was available for a specific radionuclide (see supplemental Tables S4-S10), that radionuclide was not measured on that system. One system (E1) appeared defective as it systematically underestimated the activity (typically by more than 10%) of all samples (see Fig. 1). Therefore, this system was excluded from further analysis. This resulted in a total of 745 activity measurement datasets for further analysis.

**Fig. 2:**
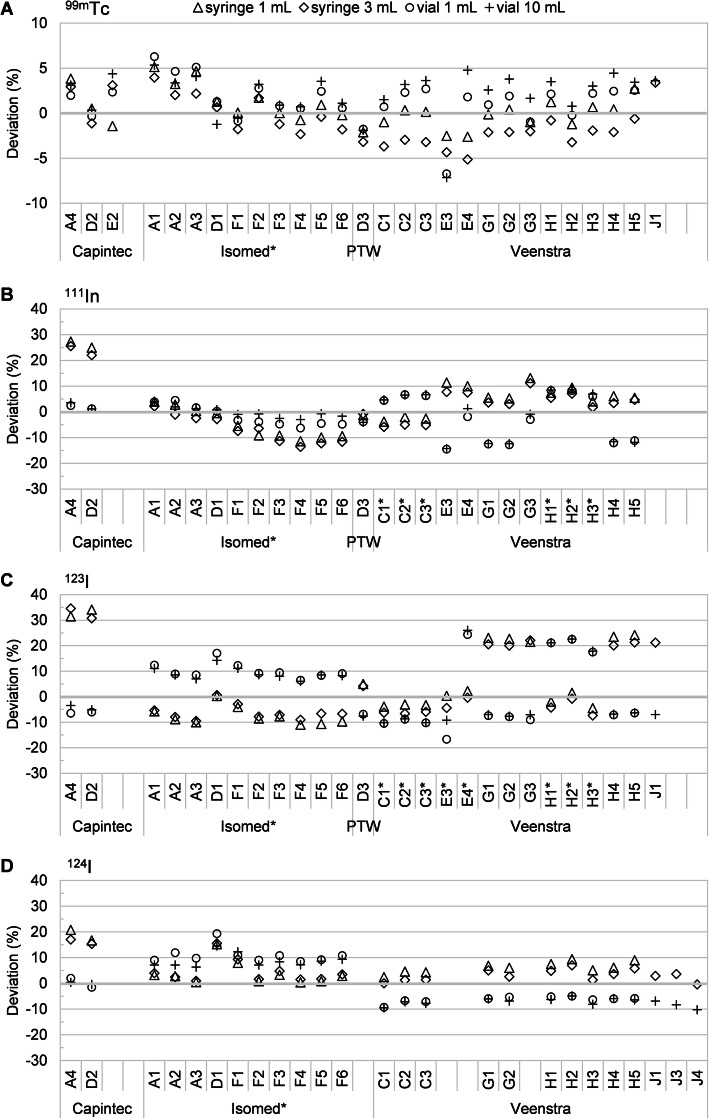
Percentage deviations of all the activity measurements used for analysis, for each system tested, for the diagnostic radionuclides. **a** ^99m^Tc, **b** ^111^In, **c** ^123^I, **d** ^124^I. Systems using sample geometry calibration/correction factors are labeled with an asterisk (*)

**Fig. 3:**
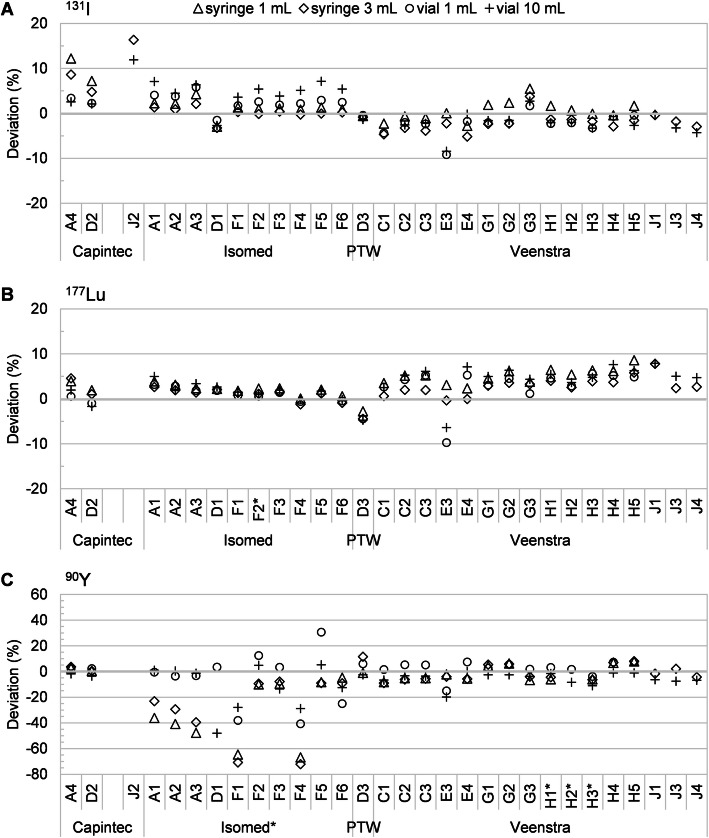
Percentage deviations of all the activity measurements used for analysis, for each system tested, for the therapeutic radionuclides. **a** ^131^I, **b** ^177^Lu, **c** ^90^Y (data not shown: D1 syringe 1 mL 158.2%, D1 syringe 3 mL 94.0%, H2 syringe 1 mL 423.9%, H2 syringe 3 mL 383.6%). Systems using sample geometry calibration/correction factors are labeled with an asterisk (*)

An overview of the intercomparison results is provided in Fig. 1 as box-whisker plots of the percentage deviations from all analyzed radionuclide calibrator measurements. Figures 2 and 3 show the individual percentage deviations grouped per manufacturer (excluding defective/invalid measurements), for the diagnostic and therapeutic radionuclides, respectively. Table 2 indicates the percentage of activity measurements that exceeded a given range of deviation from the reference activity.

**Table 2:** Percentage of activity measurements that exceed a given deviation from the reference activity

Radionuclide	Sample type	Deviating measurements (%)		No. of measurement datasets	No. of systems tested
		± 5%	± 10%		
^99m^Tc	Syringes	3.6	0.0	55	28
	Vials	9.1	0.0	55	28
^111^In	Syringes	61.5	25.0	52	26
	Vials	40.4	19.2	52	26
^123^I	Syringes	67.9	34.0	53	27
	Vials	98.1	35.8	53	27
^124^I	Syringes	36.2	12.8	47	25
	Vials	89.4	17.0	47	25
^90^Y	Syringes	74.5	27.3	55	29
	Vials	47.3	23.6	55	29
^131^I	Syringes	10.7	3.6	56	30
	Vials	18.2	1.8	55	29
^177^Lu	Syringes	18.2	0.0	55	29
	Vials	29.1	0.0	55	29

### Diagnostic radionuclides

#### ^99m^Tc

For ^99m^Tc, only 6% (7/110) of all measurements were not within ± 5% of the reference value. No dataset showed deviations larger than ± 10%. For all sample configurations, the median deviation was within 3.2% from the reference value and there was little spread in measurement deviations (largest IQR 4%), indicating a good and reproducible measurement accuracy for ^99m^Tc.

With a median difference of less than ± 2% in measurement deviations between syringes and vials (IQR 3%), the dependency on container type was mostly small.

#### ^111^In

A substantial amount of the ^111^In measurements did not meet the recommended accuracy of ± 5% (51%; 53/104), nor the less strict limit of ± 10% (22%; 23/104). Although the median deviation of all systems was within 3.5% from the reference value for all sample types, the IQR ranged up to 12%.

Additionally, the measurement accuracy often depended on sample container, with a median difference between syringes and vials of ± 8% (IQR 14%). Typically, this was most pronounced for systems that did not incorporate any correction for measurement geometry (i.e., Capintec systems, D3, E3, E4, G1–G3). However, even systems with sample geometry calibration/correction settings were not always accurate within ± 5% or ± 10% (Isomed F3–F6).

#### ^123^I

The majority of the ^123^I measurements did not meet the recommended ± 5% accuracy limit (83%; 88/106). Moreover, a substantial amount of measurements did not meet the ± 10% limit either (35%; 37/106). For all the samples, the median deviation of all systems was within 7.4% from the reference value, and the largest IQR was 30%. Furthermore, we observed a large dependence on sample type with a median difference between syringes and vials of ± 17% (IQR 16%).

Typically, systems without sample geometry corrections tended to overestimate the activity in syringes but underestimate the activity in vials, whereas the opposite trend was observed for systems that did incorporate sample geometry corrections.

#### ^124^I

A substantial amount of the ^124^I measurements did not meet the recommended ± 5% (63%; 59/94) nor the less strict limit of ± 10% (15%; 14/94). For all the samples, the median deviation of all systems was within 4.9% from the reference value, and the largest IQR was 16%. Additionally, with a median difference between syringes and vials of ± 10% (IQR 8%), ^124^I showed a substantial sensitivity to sample geometry. Syringe measurements showed a rather small overestimation in measured activity (largest median deviation of 4.8%) with a relatively small IQR (maximum 6%). For vials, however, the accuracy typically depended on whether the system used sample-specific calibration/correction settings (median deviation of all vial measurements of 9.1%) or not (− 6.3%).

### Therapeutic radionuclides

#### ^131^I

For ^131^I, 14% (16/111) and 3% (3/111) of all activity measurements were not within ± 5% and ± 10% of the reference values, respectively. For all the samples, the median deviation of all systems was within 1.1% from the reference value, and the largest IQR was 7%. Furthermore, with a median difference of less than ± 2% between the deviations of syringes and vials (IQR 3%), sample geometry effects were mostly small.

#### ^177^Lu

A substantial amount of all ^177^Lu measurements did not meet the recommended ± 5% (24%; 26/110) criterion. However, no dataset showed deviations exceeding the ± 10% limit. For all the samples, the median deviation was within 3.7% from the reference value. All IQR values were within 4%, indicating a fair to good reproducible measurement accuracy. Moreover, with a median difference of approximately ± 1% between the deviations of syringes and vials (IQR 2%), sample geometry effects were small.

#### ^90^Y

The majority of the ^90^Y measurements did not meet the recommended ± 5% accuracy limit (61%; 67/110). Moreover, a substantial amount of measurements did not meet the ± 10% limit (26%; 28/110). We observed a large variability in measurement accuracy depending on the system (type) and manufacturer.

Isomed systems, using specific calibration settings for each sample configuration, often showed very large underestimation (> 30%) of the ^90^Y reference activity, most pronounced for syringes, with IQR values up to 45%. Additionally, we found a large variability in performance between systems of the same type using identical calibration factors (e.g., A1 vs F1). Moreover, with a median difference between the deviations for syringes and vials of ± 33% (IQR 30%), geometry effects were very large.

Instead, the other radionuclide systems typically performed better, particularly for vials. For all sample configurations, the mean deviations were within 3.5%, and the largest IQR was 12%. With a median difference in measurement deviations between syringes and vials of ± 6% (IQR 8%), geometry effects were much smaller compared to the Isomed systems.

Interestingly, two systems resulted in unexpectedly high deviations from the reference activity: Isomed D1 (maximum deviation 158%) and Veenstra H2 (maximum deviation 424%).

### Theragnostic pairs

**Fig. 4:**
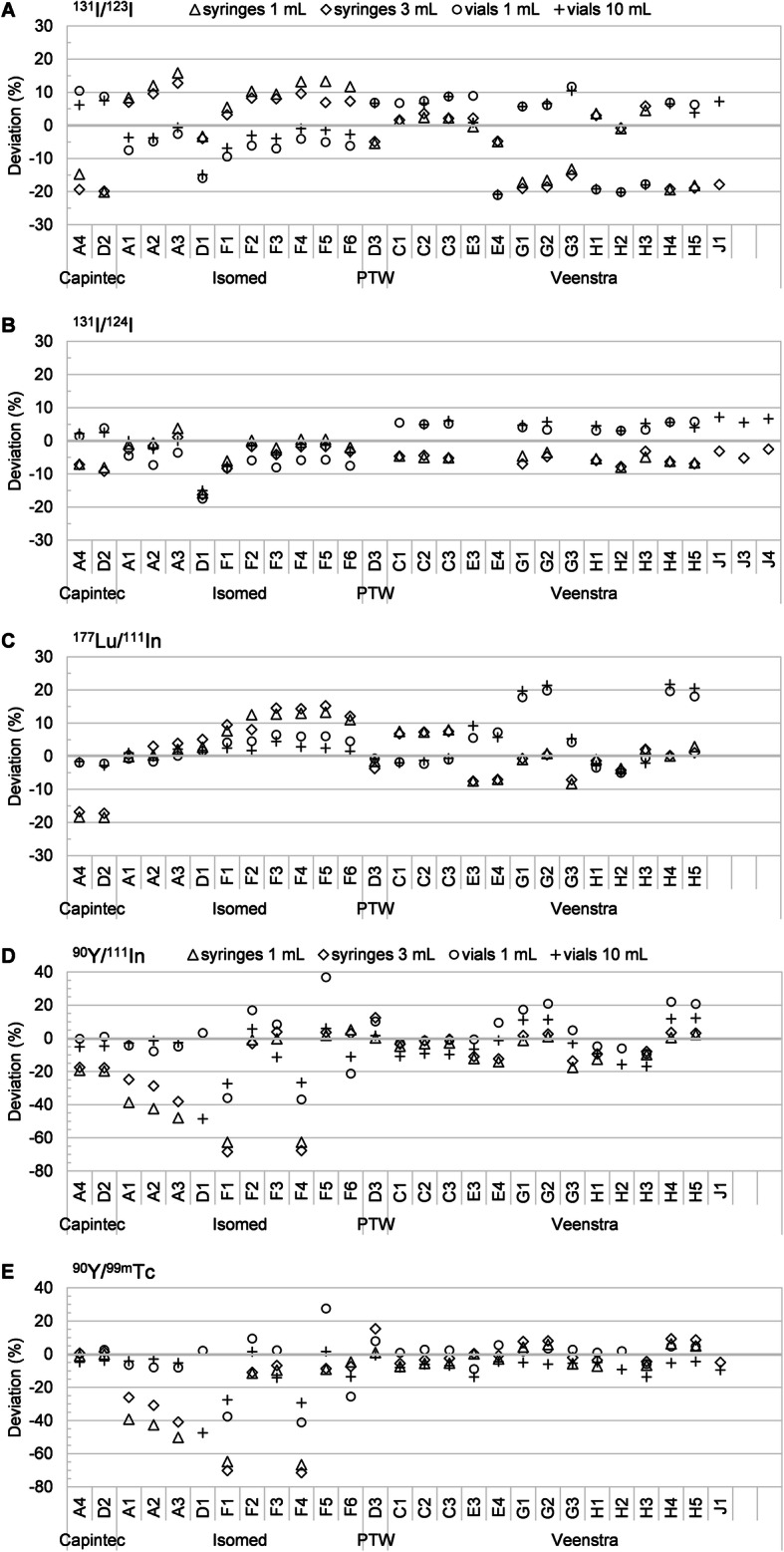
Percentage combined deviations for the theragnostic radionuclide pairs considered, when both radionuclides are measured on the same device and using the same sample geometry. **a** ^131^I/^123^I, **b** ^131^I/^124^I, **c** ^177^Lu/^111^In, **d** ^90^Y/^111^In, **e** ^90^Y/^99m^Tc

Figure 4 shows the combined systematic percentage deviations for the theragnostic pairs considered (^131^I/^123^I, ^131^I/^124^I, ^177^Lu/^111^In, ^90^Y/^99m^Tc, ^90^Y/^111^In), when both radionuclides are measured on the same device with the same sample geometry.

The combined deviations of the theragnostic pairs show substantial variability in measurement accuracy between systems and manufacturers with a dependency on calibration/correction setting and sample geometry. Generally speaking, roughly half of all investigated theragnostic combinations would introduce a bias in the therapeutic dose larger than ± 5%, and for one quarter of these combinations in a bias larger than ± 10% (Table 3). This performance is even worse when activity measurements in different containers are combined: of all administrations, two thirds would introduce a bias larger than ± 5% and one third larger than ± 10% (data not shown).

**Table 3:** Percentage of theragnostic activity measurements that exceed a given deviation from the reference activities

Theragnostic pair	Sample type	Deviating measurements (%)			No. of pairs of measurement datasets	No. of systems tested
		± 5%	± 10%	± 20%		
^131^I/^123^I	Syringes	64.2	41.5	1.9	53	27
	Vials	75.0	25.0	7.7	52	26
^131^I/^124^I	Syringes	44.7	4.3	0.0	47	25
	Vials	47.8	4.3	0.0	46	24
^177^Lu/^111^In	Syringes	55.8	25.0	0.0	52	26
	Vials	30.8	15.4	5.8	52	26
^90^Y/^99m^Tc	Syringes	67.9	32.1	26.4	53	27
	Vials	52.8	20.8	13.2	53	27
^90^Y/^111^In	Syringes	57.7	50.0	26.9	52	26
	Vials	65.4	42.3	19.2	52	26

## Discussion

Administering the correct amount of therapeutic activity to patients is of utmost importance in personalized molecular radiotherapy. Typically, (inter)national guidelines recommend stricter accuracy demands (± 5%) for therapeutic than for diagnostic radionuclides (± 5–10%) [6–8]. However, in case of theragnostics, where the therapeutic activity is optimized based on pre-therapeutic dosimetry/uptake calculations using diagnostic imaging, accurate quantification of the diagnostic activity is of equal importance as accurate therapeutic activity quantification. Therefore, to prevent introducing a substantial error in the therapeutic doses delivered to patients, we advocate to apply the ± 5% accuracy limit also for diagnostic radionuclides in a theragnostic setting.

In our study, we found one radionuclide calibrator (E1) that showed large deviations (> 10% underestimations) for all radionuclides, therefore appearing to be malfunctioning. This system was recently installed and was not yet (fully) validated nor released for clinical use. These observations indicate that extensive validation of all clinically used radionuclides is of vital importance.

### Individual radionuclides

This intercomparison shows that radionuclide calibrator measurements of ^99m^Tc, still the workhorse of nuclear medicine, are (nearly) always correct, in agreement with values reported in literature [9, 14]. The same cannot be said for the other diagnostic radionuclides evaluated. For ^111^In, ^123^I, and ^124^I, measurement deviations frequently exceeded the ± 5% and often even the ± 10% limits. This is in agreement with values reported in literature for ^111^In and ^123^I [9, 10, 12]. To the best of our knowledge, no multi-center data are available on the typical accuracy of ^124^I clinical activity measurements. In particular, these radionuclides (^111^In, ^123^I, and ^124^I) show a large dependence on sample geometry (particularly sample container) caused by self-absorption of the emitted low-energy X-rays within the sample itself. Consequently, accurate activity measurement of these radionuclides requires specific calibration or correction factors for the sample geometry [22, 23]. When factory settings dedicated to specific sample configurations are available, they must be experimentally verified prior to clinical use, as they might not be accurate for the specific containers used locally. This was the case for many activity measurements of ^123^I, ^111^In, and ^124^I. Alternatively, selective absorption of low-energy X-rays using a copper/aluminum filter is an effective method to minimize the variability in activity measurements caused by sample geometry [23, 24]. In this intercomparison, a copper filter was available for two systems, but appropriate calibration factors for measurements with filter had yet to be determined.

Regarding the therapeutic radionuclides, ^177^Lu measurements were almost always within ± 5% from the reference activity, and never deviated by more than ± 10%, in agreement with values previously reported for Capintec systems [13]. A tendency to overestimate the reference activity values by typically a few percent was observed, which might (partially) be attributed to the calibrators being sensitive to the presence of the ^177m^Lu impurity. Our study presents new data for ^177^Lu, particularly on the accuracy of medical calibrators from different suppliers, and using clinical sample configurations. Similar as for ^177^Lu, the majority of calibrators were accurate for measuring ^131^I albeit with a slightly higher deviation (sometimes > ± 5%, rarely > ± 10%). This is in agreement with values reported in literature [15]. In contrast, for ^90^Y, some systems showed incorrect measurements to an unacceptable level: the deviation ranged from a 72% underestimation to a 424% overestimation. Indeed, in literature, large measurement errors up to ± 50% have been reported [13]. In particular, although all Isomed devices used factory-set corrections for sample geometry, they were highly sensitive to the sample container and volume of solution and large measurement deviations were observed. Also, two systems (D1 and H2) showed extremely high overestimations for the syringe measurements, but not for the vials. Interestingly, this effect was not observed for other systems of the same type and with the same (factory-set) calibration factors. Most likely, in these two systems, high-energy beta radiation was able to reach the ionization chamber in the syringe samples but not in the vial samples. Indeed, the radionuclide calibrator response to high-energy beta particles is highly sensitive to even small variations in the material and design specifications of the measurement set-up [4]. This clearly indicates the importance of extensive validation of each individual system for each radionuclide and clinically used sample geometry.

### Theragnostic applications

The present study sets the first reference on typical combined errors associated to clinical radiopharmaceutical activity measurements in a theragnostic setting. Considering 5 clinically relevant theragnostic pairs (^131^I/^123^I, ^131^I/^124^I, ^177^Lu/^111^In, ^90^Y/^99m^Tc, ^90^Y/^111^In), this intercomparison study showed that poor accuracy in radionuclide calibrator activity measurements of therapeutic and diagnostic radionuclides can introduce a relatively large (> ± 10%) bias in the therapeutic doses delivered to patients in theragnostic applications. Such errors should be minimized as much as practically possible, therefore the recommendation to apply a standard ± 5% accuracy limit to calibrator activity measurements of both therapeutic and diagnostic radionuclides.

The best way to limit the error in the administration of activity is to ensure accurate and reproducible activity measurements of both radionuclides involved in the theragnostic application. This can be achieved by proper evaluation of the accuracy of the measurement settings of the calibrators for the radionuclides and sample configurations found in clinical practice, together with an assessment of other sources of uncertainty in the activity measurements and proper maintenance through a quality assurance program [6]. These procedures may lead to re-calibration of the device or determination of appropriate correction factors, and optimization of the source configurations (e.g., choice of container) or other measurement settings or procedures used for activity measurements. After all, the error in the assessment of patient administered activities is only one of the several sources of uncertainty in the dosimetry process [25]. Minimizing its contribution to the overall uncertainty is the best starting point towards patient treatment optimization in molecular radiotherapy.

### Uncertainties in the clinical activity measurements of this study

As reported in detail by Gadd et al. [5], radioactivity measurements using radionuclide calibrators are affected by different sources of uncertainty, including the accuracy of calibration factors, sample geometry effects, photon-emitting radionuclide impurities, background variability, system non-linear response, short-term response variability, reproducibility of sample position, and influence of external shielding. These uncertainty components are dependent on the specific measurement set-up (calibrator unit and its accessories, shielding, local background field), the radionuclide, and/or the level of activity (ionization current) being measured.

In this study, the clinical measurement accuracy of radionuclide calibrators was tested for 7 radionuclides used in theragnostics, each in 4 sample configurations.

The effect of the sample type of container (syringe vs vial) was evaluated. As previously addressed, this effect was a significant source of variability in the activity measurements of all the radionuclides, with the magnitude of the effect (median) being large (> ± 5%) for ^90^Y, ^123^I, ^111^In, and ^124^I; mostly small (± 2%) for ^131^I and ^99m^Tc; and small (± 1%) for ^177^Lu.

The influence of the short-term response variability in the activity measurements was reduced by taking the average of three consecutive activity readings. Although the measurement statistical uncertainty *u*_*m*_ was within 0.7% for the large majority (> 75%) of the activity datasets, which indicates a good short-term measurement reproducibility, it is not negligible and in a clinical setting (where an average value is generally not estimated) would cause a spread in the activity assessment.

The background reading was subtracted from all activity measurements. Yet, the uncertainty due to background variability was not assessed. This uncertainty can have an important bearing in the measurement of low activities and radionuclides with a low response per unit activity, such as ^90^Y. In this study, the highest background-to-sample reading ratios were obtained, as expected, with the vials with 1 mL (samples with low activity), and were ≤ 3.7% for ^90^Y, 1.7% for ^177^Lu, and 0.9% for the other radionuclides. For the vials filled with 10 mL (samples with the highest activity), background fractions were considerably lower (less or equal to 0.6% for ^90^Y and 0.2% for the other radionuclides). Assuming a high uncertainty of 10% in the background measurement, the potential error introduced in the estimated net activities of the low-activity vial samples of this study would be ≤ ± 0.38% (^90^Y), ± 0.17% (^177^Lu), and ± 0.09% (other radionuclides). Although such potential error is not negligible for ^90^Y and ^177^Lu, it is much lower compared to the measurement deviations observed in this intercomparison for the vial and syringe samples with 1–3 mL, suggesting that it is not the main cause of the spread in ^90^Y and ^177^Lu measurements of the samples with the lowest activities. For the other samples and radionuclides, the potential error from the background uncertainty is negligible.

All radionuclide solutions were checked for the presence of photon-emitting impurities by high-resolution gamma spectrometry. Impurities were detected only in ^123^I (^125^I), ^124^I (^125^I), and ^177^Lu (^177m^Lu). From these impurities, only the ^177m^Lu impurity has a significant effect on activity measurements in a radionuclide calibrator (0.51% overresponse for the Fidelis). Since the activities measured with the hospital calibrators were not corrected for this effect, this remains a source of uncertainty in the ^177^Lu intercomparison results.

Information regarding other sources of uncertainty was not gathered from the participating hospitals. Yet, hospitals were encouraged to make a more detailed uncertainty assessment for their activity measurements, since this is essential to evaluate the agreement with the reference values and determine which corrective actions are needed to improve the accuracy and reliability of their activity measurements. In general, that assessment should be within the practical reach of hospitals, since most of the sources of error mentioned above can be quantified by following a thorough quality control program [5, 6, 8].

### Study limitations

It should be noted that not all the calibrator systems tested were clinically used to measure all the radionuclides considered in this study. Since hospitals may validate a device only for the specific radionuclides used in their clinical practice, some specific results of this study may not fully represent the local (hospital) measurement capability.

Clinical activity measurements can bear additional uncertainties beyond those accounted in this study. The amounts of activities administered to patients in nuclear medicine theragnostics are in the range of tens to several hundred megabecquerels for imaging studies and a couple to several gigabecquerels for therapeutic purposes, whereas in this study the sample activities were in the range of 4–162 MBq for diagnostic radionuclides and 9–312 MBq for therapeutic radionuclides (see values per radionuclide in Table 1). Linearity effects, which are typically in the range of ± 1% to few percent [3, 5], become more important for the much broader range of activities measured in clinical applications. Also, in clinical practice, therapeutic and diagnostic radionuclides are often not measured using the same (sample) measurement geometry. For instance, ^90^Y is often assayed using manufacturer-supplied vials and/or acrylic shields. Indeed, the (combined) errors in theragnostic activity measurements will depend on the specific measurement settings used for each radionuclide. Moreover, the response of a radionuclide calibrator to ^90^Y also depends on the physicochemical form of the ^90^Y compound [26]. In this study, ^90^Y samples were prepared based on a ^90^Y chloride aqueous solution. Yet, in liver radioembolization procedures, which represent the main clinical application of the theragnostic pair ^90^Y/^99m^Tc, ^90^Y is administered to patients in the form of suspensions of resin/glass microspheres. Activity measurements of ^90^Y microspheres may require the use of different calibration factors and present further challenges whose associated errors might not be reflected in the overall measurement performance obtained here using ^90^Y chloride.

## Conclusion

This intercomparison showed that, while ^99m^Tc, ^131^I, and ^177^Lu activity measurements are mostly accurate, there is still significant room for improvement for ^111^In, ^123^I, ^124^I, and ^90^Y. For these radionuclides, the radionuclide calibrator response is particularly sensitive to the sample and detector geometry. Consequently, substantial over- or underdosing (> ± 10%) of therapeutic administrations is likely to occur in a theragnostic setting. A key message from this intercomparison is that, prior to clinical release, radionuclide calibration factors and sample geometry correction factors should be verified for each radionuclide and sample configuration used in practice. A unified international standard for testing and calibrating medical radionuclide calibrators is pressingly needed to boost the implementation of quantitative accuracy in nuclear medicine theragnostics.

## Supplementary Information

**Additional file 1.** Supplemental data

## Data Availability

The data that support the findings of this study are available from the corresponding author RW upon reasonable request and with permission of the institution where measurement data was acquired.

## Abbreviations

IQR: Inter-quartile range
JRC: Joint Research Centre
NPL: National Physical Laboratory
PET: Positron emission tomography
SCK CEN: Belgian Nuclear Research Centre
SD: Standard deviation
SPECT: Single photon emission computed tomography
