# Supplementary material for: An international multi-center investigation on the accuracy of radionuclide calibrators in nuclear medicine theragnostics
Source: EJNMMI Phys. 2020 Nov 23;7:69. doi: 10.1186/s40658-020-00338-3 (PMC7683758; doi:10.1186/s40658-020-00338-3)
Supplement: Supplementary file 1 — Additional file 1. Supplemental data [file 40658_2020_338_MOESM1_ESM.docx]

**Supplemental data**

**Evaluation of radionuclidic impurities**

Each stock solution was checked for the presence of gamma-ray emitting radionuclide impurities by gamma-ray spectrometry analysis using a high-purity germanium detector (Canberra HPGe GR1018; Mirion Technologies, Georgia, USA), calibrated conform the procedures described by the International Atomic Energy Agency (IAEA technical reports series no 454, 2006). From each stock solution, three aliquots were prepared by pipetting 20 µL of solution into a 1.5-mL Eppendorf cup. Each aliquot was measured twice at a fixed distance of 18.5 cm from the detector cap in order to minimize system dead-time effect. The first (early) gamma spectrometry measurement, having a measurement time of 5 min, was performed at the day of sample preparation to verify the absence of short-lived radionuclide impurities. The second (late) measurement, having a measurement time of 30 min, was performed after a minimum of 10 half-lives of the main radionuclide (6.5 for ^131^I, 8 for ^177^Lu) to check for short and long-lived radionuclide impurities, respectively. Each measurement was corrected for radioactive decay, dead-time, background and true coincidence effects. For each stock solution, the radionuclide impurities were determined as the average values of the three aliquots measured.

Additionally, the minimum detectable activities (MDA) of potential long-lived radionuclidic impurities (^99^Mo for ^99m^Tc, ^114m^In for ^111^In, ^121^Te and ^125^I for ^123^I, ^125^I for ^124^I and ^131^I, ^88^Y for ^90^Y) were estimated, to assess the potential effect that these impurities would have on the response of a radionuclide calibrator, in the hypothetical situation that they were present in the main radionuclide solution but could not be detected by the gamma spectrometry measurements. MDA values were calculated following the calculation method of ISO-11929-3 standard [17] for a measurement time of 30 min (corresponding to the late gamma spectrometry measurement) and a significance level of 95%. The effect of impurities on radionuclide calibrator measurements was assessed for a Fidelis secondary standard calibrator.

The (hypothetical) maximum impurity levels obtained are specified in Table S1, for the detected impurities and for the potential impurities not detected. Trace amounts of ^125^I in ^123^I and ^124^I, and of ^177m^Lu in ^177^Lu, were detected. From these detected impurities, only the impurity level of ^177m^Lu (in ^177^Lu) has a significant effect on radionuclide calibrator measurements of the main radionuclide (0.51% over-response for a Fidelis chamber). The influence of the ^125^I impurities on ^123^I and ^124^I calibrator activity measurements is negligible (see Table S1). On the other hand, from the impurities that were not detected, only the hypothetical impurity levels of ^88^Y (in ^90^Y) and ^99^Mo (in ^99m^Tc) would have a non-negligible effect on calibrator measurements (see Table S1). Nevertheless, these potential effects can be considered small (< 0.5%).

***Table S1:*** *Gamma spectrometry minimum detectable activities (MDA), maximum fraction of impurity in main radionuclide, and estimated effect on the response of the Fidelis reference chamber, for (potential) photon-emitting radionuclidic impurities.*

| Main radionuclide | Impurity radionuclide | Impurity MDA (Bq) | Max. fraction of potential impurity (%) | Max. fraction of detected impurity (%) | (Potential) over-response of Fidelis (%) |
| --- | --- | --- | --- | --- | --- |
| ^99m^Tc | ^99^Mo | 130 | 0.048 | - | (0.10) |
| ^111^In | ^114m^In | 15 | 0.021 | - | (< 0.01) |
| ^123^I | ^121^Te | 2 | 0.0018 | - | (< 0.01) |
| ^123^I | ^125^I | 2 | 0.0009 | 0.030 | < 0.01 |
| ^124^I | ^125^I | 2 | 0.0021 | 0.037 | < 0.01 |
| ^131^I | ^125^I | 2 | 0.0018 | - | (< 0.01) |
| ^177^Lu | ^177m^Lu | 7 | 0.0044 | 0.017 | 0.51 |
| ^90^Y | ^88^Y | 6 | 0.0016 | - | (0.49) |

**Determination of reference activity**

The activity measurements performed with the reference chambers (Fidelis and ISOCAL-III) were corrected for linearity, detected radionuclide impurities (significant only for (^177m^Lu/)^177^Lu measurements) and for deviations in response against the NPL master chamber (see Table S2). For the latter correction, radionuclide- and chamber-dependent correction factors were estimated from the NPL acceptance testing data of each system (NPL certificates) complemented with simulation data on the energy dependence of a Vinten chamber [21], as follows. For the gamma emitters (^99m^Tc, ^111^In, ^123^I, ^124^I, ^131^I, ^177^Lu), correction factors were calculated from a second-order polynomial curve fit of the experimental Fidelis-NPL (or ISOCAL-NPL) response ratios of 4 (or 3) long-lived test sources (^241^Am, ^57^Co, ^137^Cs and ^60^Co for the Fidelis; ^241^Am, ^137^Cs and ^60^Co for the ISOCAL) as a function of the mean energy of the radionuclide photon emissions (calculated as the weighted sum of the photon energy, emission yield and simulated detection efficiency). For ^90^Y measurements with the Fidelis only, the correction factor was estimated based on the experimental Fidelis-NPL ratio of a ^90^Sr/^90^Y source (2 mL liquid in a 10 mL P6 vial), and the difference in the simulated chamber response of a ^90^Sr/^90^Y source and a ^90^Y source.

***Table S2:*** *Corrections applied to activity measurements performed with the reference chambers (Fidelis and ISOCAL-III).*

| Correction | Fidelis | ISOCAL-III |
| --- | --- | --- |
| Linearity | < +0.4% (dependent on measured current) | none |
| Radionuclide impurities | ^177^Lu: -0.5% (^177m^Lu) | ^177^Lu: -0.5% (^177m^Lu) |
| Relative response against NPL master chamber | gamma emitters: ≤ -1.1% | gamma emitters: ≤ -0.6% |
|  | ^90^Y: -14.5% |  |

***Table S3:*** *Uncertainty assessment of radionuclide activity measurements using the reference chambers (Fidelis and ISOCAL-III).*

| Source of uncertainty | Standard uncertainty (*k*=1) (%) |
| --- | --- |
| NPL calibration factor | ^99m^Tc: 0.90, ^111^In: 0.75, ^123^I: 0.90, ^124^I: 0.50,  ^131^I: 0.40, ^177^Lu: 0.50, ^90^Y: 2.17 |
| Energy response correction | gamma emitters: 0.27, ^90^Y: 2.0 |
| Day-to-day system stability | Fidelis: ≤ 0.1, ISOCAL: ≤ 0.2 |
| Linearity (correction) | Fidelis (linearity correction): 0.03, ISOCAL: 0.1 |
| Counting statistics of ionization current | Fidelis: ≤ 0.05, ISOCAL: ≤ 0.7 (^90^Y excluded) |
| Geometry effects for ^124^I meas. in Schott 1+ vial (this study) vs P6 vial (NPL calibration factor) | ^124^I: 0.6 |

**Radionuclide calibrator measurement settings**

***Table S4:*** *Radionuclide calibrator specifications.*

| Manufacturer | Model (Chamber) | University hospital | Radionuclide calibrator | Year of purchase |
| --- | --- | --- | --- | --- |
| Capintec | CRC-25R | A | 4 | 2010 |
| Capintec | CRC-15R | D | 2 | 1995 |
| Capintec | CRC-25PET | E | 2 | 2018 |
| Capintec | CRC-15PET | J | 2 | 1992 |
| Nuvia | Isomed 2010 (640000-17) | A | 1 | 2015 |
| Nuvia | Isomed 2010 (640000-17) | A | 2 | 2015 |
| Nuvia | Isomed 2010 (640000-17) | A | 3 | 2015 |
| Nuvia | Isomed 2010 (638605-01)* | D | 1 | 2017 |
| Nuvia | Isomed 2010 (640000-17) | E | 1 | 2018 |
| Nuvia | Isomed 2010 (638600-17) | F | 1 | 1999 |
| Nuvia | Isomed 2010 (640000) | F | 2 | 2010 |
| Nuvia | Isomed 2010 (638600-17) | F | 3 | 2012 |
| Nuvia | Isomed 2010 (640000) | F | 4 | 2011 |
| Nuvia | Isomed 2010 (unknown) | F | 5 | 2012 |
| Nuvia | Isomed 2010 (unknown) | F | 6 | 2012 |
| PTW Freiburg | Curiementor 4 (TN33003-1,8) | D | 3 | 2006 |
| Comecer | VIK-202 | C | 1 | 2010 |
| Comecer | VIK-202 | C | 2 | 2010 |
| Comecer | VIK-202 | C | 3 | 2004 |
| Comecer | VIK-202 | E | 3 | 1994 |
| Comecer | VIK-202 | E | 4 | 2002 |
| Comecer | VIK-202 | G | 1 | 2007 |
| Comecer | VIK-202 | G | 2 | 2007 |
| Comecer | VIK-202 | G | 3 | 1987 |
| Comecer | VIK-202 | H | 1 | 2000 |
| Comecer | VIK-202 | H | 2 | 2006 |
| Comecer | VIK-202 | H | 3 | 2011 |
| Comecer | VIK-202 | H | 4 | 2017 |
| Comecer | VIK-202 | H | 5 | 2017 |
| Comecer | VIK-202 | J | 1 | 2016 |
| Comecer | VIK-202 | J | 3 | 2011 |
| Comecer | VIK-202 | J | 4 | 2015 |

* A specific ionization chamber especially designed for the institution.

**Radionuclide calibrator measurement settings**

***Table S5:*** *Radionuclide calibrator measurement settings for ^99m^Tc.*

|  | Calibration factor/dial setting (sample geometry correction factor) | | | |
| --- | --- | --- | --- | --- |
| Radionuclide calibrator | 1 mL syringe | 3 mL syringe | 1 mL vial | 10 mL vial |
| A1 | 0.128 | 0.128 | 0.129 | 0.126 |
| A2 | 0.128 | 0.128 | 0.129 | 0.126 |
| A3 | 0.128 | 0.128 | 0.129 | 0.126 |
| A4 | 80 | 80 | 80 | 80 |
| C1 | 236 | 236 | 236 | 236 |
| C2 | 236 | 236 | 236 | 236 |
| C3 | 236 | 236 | 236 | 236 |
| D1 | 0.128 | 0.128 | 0.129 | 0.126 |
| D2 | 80 | 80 | 80 | 80 |
| D3 | 1.256 | 1.256 | 1.256 | 1.256 |
| E1 | 0.128 | 0.128 | 0.129 | 0.126 |
| E2 | 90 | 90 | 90 | 90 |
| E3 | 236 | 236 | 236 | 236 |
| E4 | 236 | 236 | 236 | 236 |
| F1 | 0.128 | 0.128 | 0.129 | 0.126 |
| F2 | 0.128 | 0.128 | 0.129 | 0.126 |
| F3 | 0.128 | 0.128 | 0.129 | 0.126 |
| F4 | 0.128 | 0.128 | 0.129 | 0.126 |
| F5 | 0.128 | 0.128 | 0.129 | 0.126 |
| F6 | 0.128 | 0.128 | 0.129 | 0.126 |
| G1 | 236 | 236 | 236 | 236 |
| G2 | 236 | 236 | 236 | 236 |
| G3 | 236 | 236 | 236 | 236 |
| H1 | 236 | 236 | 236 | 236 |
| H2 | 236 | 236 | 236 | 236 |
| H3 | 236 | 236 | 236 | 236 |
| H4 | 236 | 236 | 236 | 236 |
| H5 | 236 | 236 | 236 | 236 |
| J1 | 236 | 236 | 236 | 236 |
| J2 | Not measured | Not measured | Not measured | Not measured |
| J3 | Not measured | Not measured | Not measured | Not measured |
| J4 | Not measured | Not measured | Not measured | Not measured |

***Table S6:*** *Radionuclide calibrator measurement settings for ^111^In.*

|  | Calibration factor/dial setting (sample geometry correction factor) | | | |
| --- | --- | --- | --- | --- |
| Radionuclide calibrator | 1 mL syringe | 3 mL syringe | 1 mL vial | 10 mL vial |
| A1 | 0.069 | 0.067 | 0.08 | 0.078 |
| A2 | 0.069 | 0.067 | 0.08 | 0.078 |
| A3 | 0.069 | 0.067 | 0.08 | 0.078 |
| A4 | 303 | 303 | 303 | 303 |
| C1 | 645 (81%) | 645 (81%) | 645 (100%) | 645 (100%) |
| C2 | 645 (81%) | 645 (81%) | 645 (100%) | 645 (100%) |
| C3 | 645 (81%) | 645 (81%) | 645 (100%) | 645 (100%) |
| D1 | 0.061 | 0.061 | 0.076 | 0.076 |
| D2 | 303 | 303 | 303 | 303 |
| D3 | 0.55 | 0.55 | 0.55 | 0.55 |
| E1 | 0.061 | 0.06 | 0.076 | 0.076 |
| E2 | 1† | 1† | 1† | 1† |
| E3 | 676 | 676 | 676 | 676 |
| E4 | 676 | 676 | 676 | 676 |
| F1 | 0.061 | 0.06 | 0.076 | 0.076 |
| F2 | 0.061 | 0.06 | 0.076 | 0.076 |
| F3 | 0.061 | 0.06 | 0.076 | 0.076 |
| F4 | 0.061 | 0.06 | 0.076 | 0.076 |
| F5 | 0.061 | 0.06 | 0.076 | 0.076 |
| F6 | 0.061 | 0.06 | 0.076 | 0.076 |
| G1 | 676 | 676 | 676 | 676 |
| G2 | 676 | 676 | 676 | 676 |
| G3 | 676 | 676 | 676 | 676 |
| H1 | 676 (95%) | 676 (95%) | 676 (110%) | 676 (110%) |
| H2 | 676 (95%) | 676 (95%) | 676 (110%) | 676 (110%) |
| H3 | 676 (95%) | 676 (95%) | 676 (110%) | 676 (110%) |
| H4 | 711 | 711 | 711 | 711 |
| H5 | 711 | 711 | 711 | 711 |
| J1 | Not measured | Not measured | Not measured | Not measured |
| J2 | Not measured | Not measured | Not measured | Not measured |
| J3 | Not measured | Not measured | Not measured | Not measured |
| J4 | Not measured | Not measured | Not measured | Not measured |

† Calibration factor was not present in the communication module. In this case, it appears that the system uses a calibration factor of 1 (no error warning was displayed). This measurement was excluded from the detailed analysis.

***Table S7:*** *Radionuclide calibrator measurement settings for ^123^I.*

|  | Calibration factor/dial setting (sample geometry correction factor) | | | |
| --- | --- | --- | --- | --- |
| Radionuclide calibrator | 1 mL syringe | 3 mL syringe | 1 mL vial | 10 mL vial |
| A1 | 0.066 | 0.067 | 0.095 | 0.095 |
| A2 | 0.066 | 0.067 | 0.095 | 0.095 |
| A3 | 0.066 | 0.067 | 0.095 | 0.095 |
| A4 | 277 | 277 | 277 | 277 |
| C1 | 618 (81%) | 618 (81%) | 618 (100%) | 618 (100%) |
| C2 | 618 (81%) | 618 (81%) | 618 (100%) | 618 (100%) |
| C3 | 618 (81%) | 618 (81%) | 618 (100%) | 618 (100%) |
| D1 | 0.066 | 0.067 | 0.095 | 0.095 |
| D2 | 277 | 277 | 277 | 277 |
| D3 | 0.93 | 0.93 | 0.93 | 0.93 |
| E1 | 0.066 | 0.067 | 0.095 | 0.095 |
| E2 | 1† | 1† | 1† | 1† |
| E3 | 676 (85%) | 676 (85%) | 676 (133%) | 676 (133%) |
| E4 | 676 (85%) | 676 (85%) | 676 (133%) | 676 (133%) |
| F1 | 0.066 | 0.067 | 0.095 | 0.095 |
| F2 | 0.066 | 0.067 | 0.095 | 0.095 |
| F3 | 0.066 | 0.067 | 0.095 | 0.095 |
| F4 | 0.066 | 0.067 | 0.095 | 0.095 |
| F5 | 0.066 | 0.067 | 0.095 | 0.095 |
| F6 | 0.066 | 0.067 | 0.095 | 0.095 |
| G1 | 618 | 618 | 618 | 618 |
| G2 | 618 | 618 | 618 | 618 |
| G3 | 618 | 618 | 618 | 618 |
| H1 | 618 (100%) | 618 (100%) | 618 (130%) | 618 (130%) |
| H2 | 618 (100%) | 618 (100%) | 618 (130%) | 618 (130%) |
| H3 | 618 (100%) | 618 (100%) | 618 (130%) | 618 (130%) |
| H4 | 618 | 618 | 618 | 618 |
| H5 | 618 | 618 | 618 | 618 |
| J1 | 618 | 618 | 618 | 618 |
| J2 | Not measured | Not measured | Not measured | Not measured |
| J3 | Not measured | Not measured | Not measured | Not measured |
| J4 | Not measured | Not measured | Not measured | Not measured |

† Calibration factor was not present in the communication module. In this case, it appears that the system uses a calibration factor of 1 (no error warning was displayed). This measurement was excluded from the detailed analysis.

***Table S8:*** *Radionuclide calibrator measurement settings for ^124^I.*

|  | Calibration factor/dial setting (sample geometry correction factor) | | | |
| --- | --- | --- | --- | --- |
| Radionuclide calibrator | 1 mL syringe | 3 mL syringe | 1 mL vial | 10 mL vial |
| A1 | 0.099 | 0.101 | 0.129 | 0.128 |
| A2 | 0.099 | 0.101 | 0.129 | 0.128 |
| A3 | 0.099 | 0.101 | 0.129 | 0.128 |
| A4 | 570 | 570 | 570 | 570 |
| B1 | 813 | 813 | 813 | 813 |
| C1 | 813 | 813 | 813 | 813 |
| C2 | 813 | 813 | 813 | 813 |
| C3 | 813 | 813 | 813 | 813 |
| D1 | 0.099 | 0.101 | 0.1145 | 0.128 |
| D2 | 570 | 570 | 570 | 570 |
| D3 | Not available | Not available | Not available | Not available |
| E1 | 0.099 | 0.101 | 0.129 | 0.128 |
| E2 | 1† | 1† | 1† | 1† |
| E3 | Not available | Not available | Not available | Not available |
| E4 | Not available | Not available | Not available | Not available |
| F1 | 0.099 | 0.101 | 0.129 | 0.128 |
| F2 | 0.099 | 0.101 | 0.129 | 0.128 |
| F3 | 0.099 | 0.101 | 0.129 | 0.128 |
| F4 | 0.099 | 0.101 | 0.129 | 0.128 |
| F5 | 0.099 | 0.101 | 0.129 | 0.128 |
| F6 | 0.099 | 0.101 | 0.129 | 0.128 |
| G1 | 813 | 813 | 813 | 813 |
| G2 | 813 | 813 | 813 | 813 |
| G3 | Not available | Not available | Not available | Not available |
| H1 | 813 | 813 | 813 | 813 |
| H2 | 813 | 813 | 813 | 813 |
| H3 | 813 | 813 | 813 | 813 |
| H4 | 813 | 813 | 813 | 813 |
| H5 | 813 | 813 | 813 | 813 |
| J1 | 813 | 813 | 813 | 813 |
| J2 | Not available | Not available | Not available | Not available |
| J3 | 813 | 813 | 813 | 813 |
| J4 | 813 | 813 | 813 | 813 |

† Calibration factor was not present in the communication module. In this case, it appears that the system uses a calibration factor of 1 (no error warning was displayed). This measurement was excluded from the detailed analysis.

***Table S9:*** *Radionuclide calibrator measurement settings for ^131^I.*

|  | Calibration factor/dial setting (sample geometry correction factor) | | | |
| --- | --- | --- | --- | --- |
| Radionuclide calibrator | 1 mL syringe | 3 mL syringe | 1 mL vial | 10 mL vial |
| A1 | 0.26 | 0.26 | 0.265 | 0.265 |
| A2 | 0.26 | 0.26 | 0.265 | 0.265 |
| A3 | 0.26 | 0.26 | 0.265 | 0.265 |
| A4 | 151 | 151 | 151 | 151 |
| B1 | 480 | 480 | 480 | 480 |
| C1 | 480 | 480 | 480 | 480 |
| C2 | 480 | 480 | 480 | 480 |
| C3 | 480 | 480 | 480 | 480 |
| D1 | 0.2453 | 0.2453 | 0.25 | 0.25 |
| D2 | 151 | 151 | 151 | 151 |
| D3 | 0.93 | 0.93 | 0.93 | 0.93 |
| E1 | 0.26 | 0.26 | 0.265 | 0.265 |
| E2 | 165‡ | 165‡ | 165‡ | 165‡ |
| E3 | 480 | 480 | 480 | 480 |
| E4 | 480 | 480 | 480 | 480 |
| F1 | 0.26 | 0.26 | 0.265 | 0.265 |
| F2 | 0.26 | 0.26 | 0.265 | 0.265 |
| F3 | 0.26 | 0.26 | 0.265 | 0.265 |
| F4 | 0.26 | 0.26 | 0.265 | 0.265 |
| F5 | 0.26 | 0.26 | 0.265 | 0.265 |
| F6 | 0.26 | 0.26 | 0.265 | 0.265 |
| G1 | 480 | 480 | 480 | 480 |
| G2 | 480 | 480 | 480 | 480 |
| G3 | 447 | 447 | 447 | 447 |
| H1 | 480 | 480 | 480 | 480 |
| H2 | 480 | 480 | 480 | 480 |
| H3 | 480 | 480 | 480 | 480 |
| H4 | 480 | 480 | 480 | 480 |
| H5 | 480 | 480 | 480 | 480 |
| J1 | 480 | 480 | 480 | 480 |
| J2 | 151 | 151 | 151 | 151 |
| J3 | 480 | 480 | 480 | 480 |
| J4 | 480 | 480 | 480 | 480 |

‡ System was not commissioned to measure this radionuclide, although a calibration factor was present in the communication module (no error warning was displayed). This measurement was excluded from the detailed analysis.

***Table S10:*** *Radionuclide calibrator measurement settings for ^177^Lu.*

|  | Calibration factor/dial setting (sample geometry correction factor) | | | |
| --- | --- | --- | --- | --- |
| Radionuclide calibrator | 1 mL syringe | 3 mL syringe | 1 mL vial | 10 mL vial |
| A1 | 0.44 | 0.44 | 0.44 | 0.44 |
| A2 | 0.44 | 0.44 | 0.44 | 0.44 |
| A3 | 0.44 | 0.44 | 0.44 | 0.44 |
| A4 | 150x10 | 150x10 | 150x10 | 150x10 |
| C1 | 751x10 | 751x10 | 751x10 | 751x10 |
| C2 | 751x10 | 751x10 | 751x10 | 751x10 |
| C3 | 751x10 | 751x10 | 751x10 | 751x10 |
| D1 | 0.44 | 0.44 | 0.44 | 0.44 |
| D2 | 450x10 | 450x10 | 450x10 | 450x10 |
| D3 | 4.88 | 4.88 | 4.88 | 4.88 |
| E1 | 0.44 | 0.44 | 0.44 | 0.44 |
| E2 | 1† | 1† | 1† | 1† |
| E3 | 751x10 | 751x10 | 751x10 | 751x10 |
| E4 | 751x10 | 751x10 | 751x10 | 751x10 |
| F1 | 0.44 | 0.44 | 0.44 | 0.44 |
| F2 | 0.44 | 0.44 | 0.436 | 0.436 |
| F3 | 0.44 | 0.44 | 0.44 | 0.44 |
| F4 | 0.44 | 0.44 | 0.44 | 0.44 |
| F5 | 0.44 | 0.44 | 0.44 | 0.44 |
| F6 | 0.44 | 0.44 | 0.44 | 0.44 |
| G1 | 751x10 | 751x10 | 751x10 | 751x10 |
| G2 | 751x10 | 751x10 | 751x10 | 751x10 |
| G3 | 751x10 | 751x10 | 751x10 | 751x10 |
| H1 | 751x10 | 751x10 | 751x10 | 751x10 |
| H2 | 751x10 | 751x10 | 751x10 | 751x10 |
| H3 | 751x10 | 751x10 | 751x10 | 751x10 |
| H4 | 751x10 | 751x10 | 751x10 | 751x10 |
| H5 | 751x10 | 751x10 | 751x10 | 751x10 |
| J1 | 751x10 | 751x10 | 751x10 | 751x10 |
| J2 | Not measured | Not measured | Not measured | Not measured |
| J3 | 751x10 | 751x10 | 751x10 | 751x10 |
| J4 | 751x10 | 751x10 | 751x10 | 751x10 |

† Calibration factor was not present in the communication module. In this case, it appears that the system uses a calibration factor of 1 (no error warning was displayed). This measurement was excluded from the detailed analysis.

***Table S11:*** *Radionuclide calibrator measurement settings for ^90^Y.*

|  | Calibration factor/dial setting (sample geometry correction factor) | | | |
| --- | --- | --- | --- | --- |
| Radionuclide calibrator | 1 mL syringe | 3 mL syringe | 1 mL vial | 10 mL vial |
| A1 | 0.81 | 0.67 | 1.8 | 2.07 |
| A2 | 0.81 | 0.67 | 1.8 | 2.07 |
| A3 | 0.81 | 0.67 | 1.8 | 2.07 |
| A4 | 48x10 | 48x10 | 48x10 | 48x10 |
| C1 | 902x100 | 902x100 | 902x100 | 902x100 |
| C2 | 902x100 | 902x100 | 902x100 | 902x100 |
| C3 | 902x100 | 902x100 | 902x100 | 902x100 |
| D1 | 0.81 | 0.67 | 1.8 | 2.07 |
| D2 | 48x10 | 48x10 | 48x10 | 48x10 |
| D3 | 28.95 | 28.95 | 28.95 | 28.95 |
| E1 | 0.81 | 0.67 | 1.8 | 2.07 |
| E2 | 1† | 1† | 1† | 1† |
| E3 | 902x100 | 902x100 | 902x100 | 902x100 |
| E4 | 902x100 | 902x100 | 902x100 | 902x100 |
| F1 | 0.81§ | 0.67§ | 1.8§ | 2.07§ |
| F2 | 2.13§ | 2.13§ | 3.35§ | 3.05§ |
| F3 | 0.81§ | 0.67§ | 1.8§ | 2.07§ |
| F4 | 0.81§ | 0.67§ | 1.8§ | 2.07§ |
| F5 | 0.81§ | 0.67§ | 1.8§ | 2.07§ |
| F6 | 0.81§ | 0.67§ | 1.8§ | 2.07§ |
| G1 | 902x100 | 902x100 | 902x100 | 902x100 |
| G2 | 902x100 | 902x100 | 902x100 | 902x100 |
| G3 | 902x100 | 902x100 | 902x100 | 902x100 |
| H1 | 902x100(100%) | 902x100(100%) | 902x100(92.4%) | 902x100(92.4%) |
| H2 | 902x100(100%) | 902x100(100%) | 902x100(92.4%) | 902x100(92.4%) |
| H3 | 902x100(100%) | 902x100(100%) | 902x100(92.4%) | 902x100(92.4%) |
| H4 | 902x100 | 902x100 | 902x100 | 902x100 |
| H5 | 902x100 | 902x100 | 902x100 | 902x100 |
| J1 | 902x100(100%) | 902x100(100%) | 902x100(92.4%) | 902x100(92.4%) |
| J2 | Not measured | Not measured | Not measured | Not measured |
| J3 | 902x100(100%) | 902x100(100%) | 902x100(92.4%) | 902x100(92.4%) |
| J4 | 902x100(100%) | 902x100(100%) | 902x100(92.4%) | 902x100(92.4%) |

† Calibration factor was not present in the communication module. In this case, it appears that the system uses a calibration factor of 1 (no error warning was displayed). This measurement was excluded from the detailed analysis.

§ Measurements performed with aluminum dipper.
